# Perineal Ultrasound as a Complement to POP-Q in the Assessment of Cystoceles

**DOI:** 10.1155/2014/740925

**Published:** 2014-11-27

**Authors:** Laila Najjari, Julia Hennemann, Pia Larscheid, Thomas Papathemelis, Nicolai Maass

**Affiliations:** Department of Gynecology and Obstetrics, University Hospital Aachen, 52074 Aachen, Germany

## Abstract

*Purpose*. In the present study we want to propose a classification system to quantify cystoceles by perineal ultrasound (PUS).* Materials and Methods.* 120 PUS data were analyzed measuring the distance between the lowest point of the bladder and the midpubic line (MPL) during rest and Valsalva. Results were classified into groups and compared to POP-Q using the *κ*-coefficient. Results for exact bladder position were checked for interrater reliability using ICC and Pearson's coefficient and results for classification were checked using the *κ*-coefficient. Bladder positions at rest and Valsalva were correlated with the distance between these points.* Results*. Highly significant differences concerning the position at rest and the distance between rest and Valsalva were found between the groups. For the interrater agreement, the Pearson correlation coefficient was *ρ* = 0.98, the ICC (A-1) = 0.98, and *κ* = 1.00. Comparing the classification results for POP-Q and PUS, the kappa-coefficient was *κ* = 0.65.* Conclusion*. PUS using the MPL and the classification system is a highly reliable tool for the evaluation of cystoceles. PUS shows good correlation with POP-Q. Furthermore, PUS offers a doubtless identification of the descending organ. Further studies are needed to evaluate the clinical use of the classification system proposed here.

## 1. Introduction

Pelvic organ prolapse (POP) is a very common gynecological pathology: 20% of all women undergo surgical intervention for prolapse or urinary incontinence in their life [1]. One of the most frequent forms of POP is the descending bladder, the cystocele. Since its clinical appearance varies considerably, standardized evaluation techniques are urgently demanded to establish precise indications for surgery. Guidelines regarding this issue are currently not available.

The present diagnostic gold standard is the POP-Q (Pelvic Organ Prolapse Quantification) system introduced by the International Continence Society (ICS) in 1996, which has been proved to yield highly reproducible results [2]. This method, however, is routinely used by only 40% of the ICS-members [3], suggesting that there might be even less among nonmembers. As Auwad et al. found out, the most common reason for not using POP-Q is that its use is time consuming and confusing [3]. POP-Q is a good clinical classification system, but as it does not give objective information according to anatomical structures such as the bladder position, an additional diagnostic technique would be helpful for the preoperative planning.

In the field of imaging techniques for pelvic organ prolapse, magnetic resonance imaging (MRI), dynamic cystocolpoproctography (DCP), and perineal ultrasound (PUS) are relevant diagnostic procedures. All techniques provide a benefit concerning anatomic and physiological information compared to the POP-Q evaluation. MRI, however, is an expensive diagnostic tool with extensive waiting periods, whereas DCP includes radiation. Due to the retrograde application of contrast medium, both are partly invasive, laborious, and embarrassing for the patients.

In contrast, perineal ultrasound (PUS) is gaining importance in urogynecological diagnostics. Good availability, easy handling, low cost, and good patient acceptance are some of the conveniences which have already made PUS a popular diagnostic tool, for example, in the assessment of urinary incontinence, the detection of paraurethral pathologies, and the postoperative sonographic control of TVT slings [4].

Three studies have tested this method for the quantification of pelvic organ prolapse in comparison to the POP-Q system. Dietz et al. [5] were the first to introduce a systematic approach for this purpose. They created a horizontal reference line touching the inferoposterior margin of the symphysis pubis and yielded good correlation between traditional POP-Q and PUS in 145 patients for the anterior compartment. Kluivers et al. [6] compared POP-Q measurements, a simplified staging system (I-IV), and PUS (following the approach of Dietz et al.) as to their ability to predict symptomatic prolapse in 265 women. They concluded that PUS is inferior to POP-Q and I-IV. Broekhuis et al. [7] evaluated the metrical agreement between POP-Q, dynamic MRI, and PUS (also referring to Dietz et al. for the reference line) in 97 women and found moderate to good correlation for the anterior compartment.

In the present study we used another special classification system to identify the cystoceles. We analyzed the ultrasound data of 120 women and tested the agreement with traditional POP-Q. Furthermore, we evaluated the interrater agreement of PUS in the assessment of cystoceles, which, to our knowledge, has not been studied yet.

The present study is thus intended to investigate if PUS is an adequate method to assess cystoceles. We hypothesize that the approach presented here makes PUS a reliable diagnostic tool with results which are comparable to the POP-Q system.

## 2. Material and Methods

The present study was performed according to the declaration of Helsinki and with the approval of the local ethics committee.

120 women were enrolled. All had undergone POP-Q classification and PUS in the context of our clinical routine between January 2010 and September 2011. POP-Q classification and PUS had been performed by the same experienced examiner.

For all patients, a 3D perineal ultrasound volume had been saved, both of the resting and the straining patient. These volumes were analyzed retrospectively by two independent examiners.

The measurement was performed using 4D View (version 5.0, courtesy of GE Medical, Kretz Ultrasound, Zipf, Austria).

**Figure 1:**
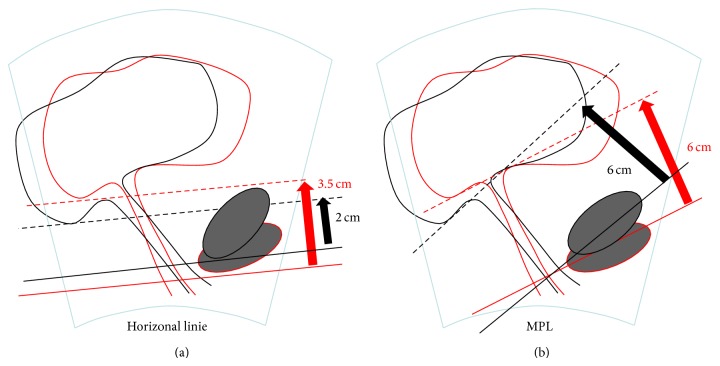
(a) Measurement as proposed by Dietz et al. [5] using a horizontal line. Original position of the probe (red): distance to the lowest point of the bladder = 3.5 cm. Shifted probe (black): distance to the lowest point of the bladder = 2 cm, resulting in 1.5 cm difference from the original position of the probe. (b) Measurement using the MPL. Original position of the probe: distance = 6 cm. Shifted probe (black): distance = 6 cm, resulting in 0 cm difference from the original position of the probe.

As mentioned above there are three previous studies according to this subject. Common to these studies is the use of a reference line crossing the inferoposterior margin of the symphysis pubis, proposed by Dietz et al. [5], as seen in Figure 1. The major disadvantage of this reference line is that it is fixed at only one point: when shifting the viewing angle by moving the probe as seen in Figure 1(a), the reference line, being oriented parallel to the lower edge of the screen, changes its angle. This causes differing results, depending on the viewing angle.

The aim of the present study is to evaluate the use of perineal ultrasound in the diagnosis of cystoceles. For the reasons listed above, we decided not to use the H-line but the midpubic line (MPL) as a reference line, already known from the MRI assessment of pelvic organ prolapse [8]. Recent studies showed that the midpubic line is a good and reliable landmark in ultrasonographic diagnostics [9]. Crossing the symphysis pubis in its longitudinal axis, it is two-point fixed (at the inferoposterior and superoanterior edge) and insensitive to changes of the viewing angle (Figure 1(b)).

**Figure 2:**
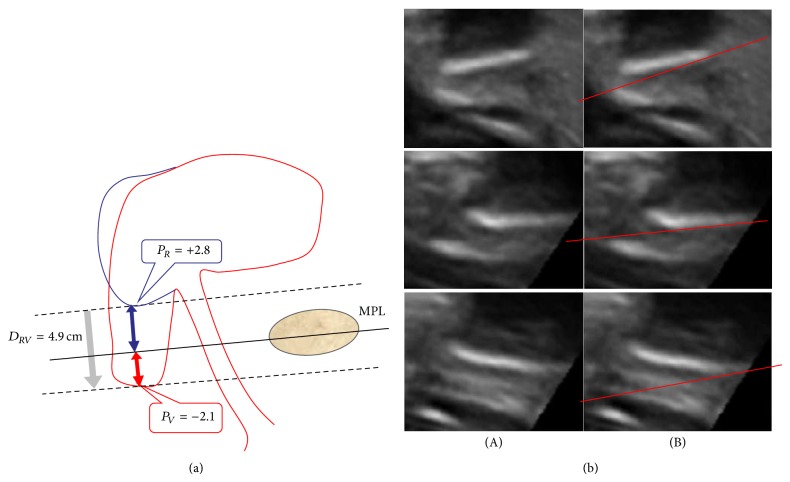
(a) Measuring the position of the lowest point of the bladder during rest (*P*_*R*_, blue) and during Valsalva (*P*_*V*_, red) in relation to the MPL.* D*_*RV*_ (bright blue arrow) equals the distance between* P*_*R*_ and *P*_*V*_, that is, the movement of the bladder from rest to Valsalva. (b) (A) Different aspects of symphysis pubis in perineal ultrasound. (b) (B) How to place the MPL when measuring* P*_*R*_ and *P*_*V*_.

As presented in Figure 2, the midpubic line (MPL) was drawn between two undulated lines indicating the two bony ends of the symphysis pubis (Figure 2(b)), thus representing a line through its longitudinal axis. A second line was drawn parallel to the MPL and moved to the lowest point of the bladder dorsal of the meatus urethrae internus. The rectangular distance between these two lines was measured, indicating the position of the lowest point of the bladder (*P*). This procedure was performed during rest (*P*_*R*_) (Figure 2(a), blue) and at the point of maximal Valsalva (*P*_*V*_) (Figure 2(a), red). The distance (*D*_*RV*_) between* P*_*R*_ and* P*_*V*_ was measured (Figure 2(a), bright blue arrow).

Even if former studies used the bladder neck as a reference point for the assessment of cystoceles, the leading edge of the bladder is used in this study, as it usually is the part provoking the symptoms and does not necessarily equal the bladder neck. Additionally, in our previous work we observed that during Valsalva the bladder neck is more difficult to assess in cystoceles than the bladder base [10].

Three methods of analysis for interrater agreement were performed: the raw values (*P*_*V*_,* P*_*R*_,* D*_*RV*_) acquired by the two examiners were correlated using Pearson's correlation coefficient and ICC (A-1). Classification results (groups I, II, and III) were compared using *κ*.

According to Altman [11], the *κ*-result should be interpreted as follows: <0.2 = poor; 0.21–0.40 = weak; 0.41–0.60 = moderate; 0.61–0.80 = good; 0.81–1.00 = excellent agreement.

**Figure 3:**
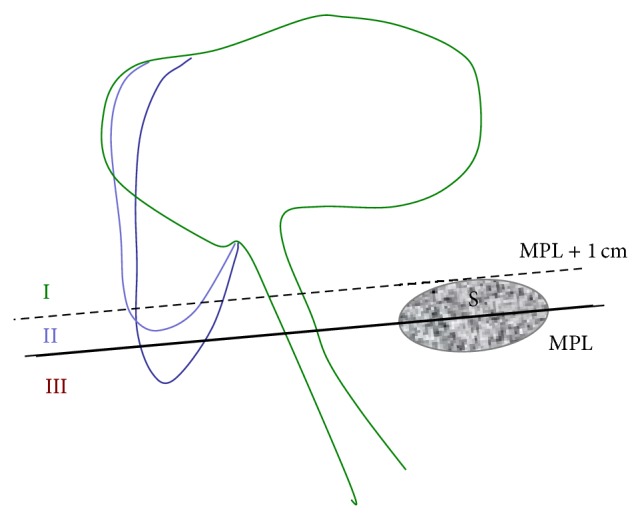
Classification system used for the present study based on the midpubic line (MPL) crossing the symphysis pubis (S) in its longitudinal axis. Bladders are assigned to groups I (>1 cm above MPL), II (<1 cm above MPL), or III (below MPL).

According to their* P*_*V*_ values, all patients were assigned to group I, II, or III (Figure 3). Means and standard deviations for* P*_*R*_ and* P*_*V*_ as well as for* D*_*RV*_, indicating the mobility of the bladder during Valsalva manoeuvre were calculated. Differences between the groups regarding* D*_*RV*_ and* P*_*R*_ were tested for significance.

The correlation between the bladder position at Valsalva (*P*_*V*_) and the extent of movement of the bladder from rest to Valsalva (*D*_*RV*_) was calculated.

As the classification system used in POP-Q and the one suggested by us for PUS are based on different measurement systems, we decided to use a common simplified classification to compare them: “normal bladder and relevant cystocele.” POP-Q stages 0 and 1 as well as PUS group I were assumed to be “normal bladders”; POP-Q stages 2–4 as well as PUS groups II and III were assumed to be “relevant cystoceles.” The statistical analysis was performed using a *κ*-analysis.

## 3. Results 

Our study yielded the following results.

Analysis of the PUS volumes was possible in all 120 patients. Their mean age was 58.2 (SD 11.3) years.

### 3.1. Interrater Agreement

For the bladder position measured at maximal Valsalva (*P*_*V*_) the Pearson correlation coefficient was *ρ* = 0.98 and the ICC (A-1) = 0.98. The kappa-coefficient indicates the chance-corrected concordance between two examiners. It was *κ* = 1.00 for the classification of the groups I, II, III.

### 3.2. Descriptive Statistics

**Figure 4:**
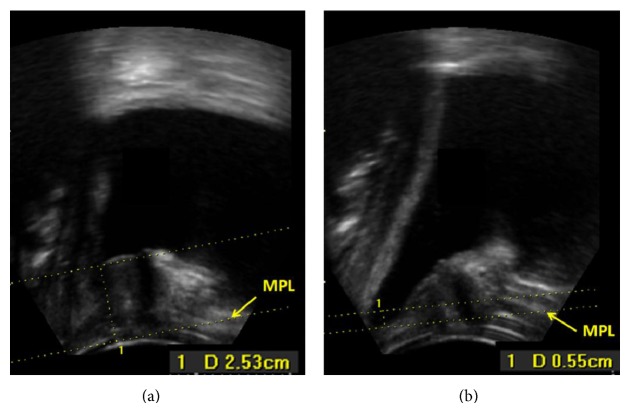
(a) Group III bladder at rest with* P*_*R*_ = 2.53 cm. “1” indicates the MPL. (b) Group III bladder at maximal Valsalva with *P*_*V*_ = −0.55 cm. “1” indicates the MPL.

Following the suggested grading system for PUS and based on the respective* P*_*V*_ values, 82 bladders were classified as group I bladders, 20 bladders as group II bladders, and 18 as group III bladders (example given in Figures 4(a) and 4(b)).

Table 1 summarizes the values of* P*_*R*_,* P*_*V,*_ and* D*_*RV*_ measured by examiner 1 (due to excellent agreement, values measured by examiner 2 are not visualized). Highly significant differences were established

- for* P*_*R*_ between groups I and II, but not between groups II and III,
- for* P*_*V*_ between groups I and II as well as between groups II and III (as* P*_*V*_ was the basis for the classification),
- for* D*_*RV*_ between groups I and II as well as between groups II and III.

For* P*_*R*_ between groups II and III, no significant differences were found.

**Figure 5:**
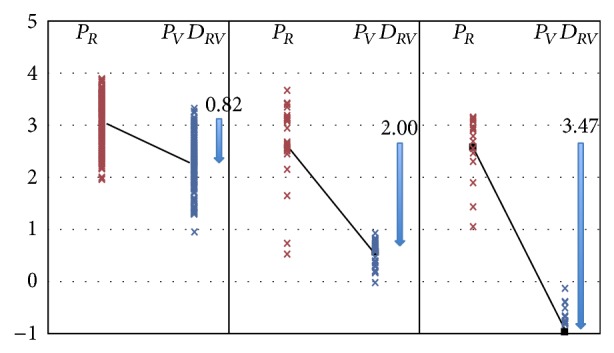
The extent of movement of group I, II, and III bladders from resting position to Valsalva position (*D*_*RV*_). Red crosses symbolize bladder positions at rest (*R*); blue crosses represent bladder positions during Valsalva (*V*). Black lines link mean positions at rest to mean positions during Valsalva. Arrows show the distance covered on average between PR and PV (*D*_*RV*_).

Figure 5 displays the extent of movement (*D*_*RV*_) of groups I, II, and III bladders from resting position (*P*_*R*_) to Valsalva position (*P*_*V*_). There are highly significant differences between all groups.

The correlation between* P*_*V*_ and the* D*_*RV*_ was *ρ* = −0.88: bladders with a low leading edge during Valsalva cover a greater distance during straining than those with a high leading edge.

### 3.3. Comparison of POP-Q Staging and PUS Staging

Comparing the classification results “normal bladder and relevant cystocele” for POP-Q staging and PUS staging, the kappa-coefficient was *κ* = 0.65.

## 4. Discussion

The results of the present study confirm our hypothesis that PUS is a reliable tool in the assessment of cystoceles and that its results are comparable to the POP-Q system.

The excellent interrater agreement of our study suggests that the use of the MPL as a reference line yields highly reproducible results which are superior to those achieved using the H-line proposed by Dietz et al. [5]. We attribute this to the fact that the MPL—unlike the H-line—is fixed at two bony points.

In order to define a clinically relevant classification system for descending bladders, we considered the anatomic landmark at which a cystocele begins to cause symptoms. Recent literature provides differing opinions regarding this issue. Some authors suggest for example the hymen as a possible landmark (Kluivers et al. [6]). To provide a cut-off for symptomatic cystoceles, a landmark at 1 cm proximal to the hymen was chosen. As the MPL crosses the hymenal remnants in the sagittal view [8], we decided to set up the following classification to describe the bladders seen in our study: we defined the three groups the volumes were assigned to and made the following observations.

Group I bladders were defined to have their lowest point during Valsalva (*P*_*V*_) at more than 1 cm above the MPL, corresponding to the hymenal remnants. As 87% of group I bladders were classified as stage 0 or 1 in POP-Q, we assume that these bladders should not be considered as descending.

Group II bladders were defined to have their* P*_*V*_ at up to 1 cm above the MPL, but still above the MPL, suggesting that the prolapse might be seen during inspection and maybe also felt by the coughing patient. Group II bladders descend significantly deeper than group I bladders. These points lead us to the conclusion that group II bladders may already be characterized as descending bladders.

Group III bladders were defined to have their* P*_*V*_ below the MPL and are thus visible during inspection and felt by the patient when she is straining or coughing [6]. Additionally, group III bladders move significantly more than group II bladders from rest to Valsalva. These facts suggest that, based on clinical and sonographic findings, these bladders should definitely be regarded as cystoceles.

Furthermore, we have determined the interrater agreement for the assessment of cystoceles using PUS. The metrical correlation was excellent; the classification agreement (group I, II, or III bladder) was “perfect” [11]. As far as we are aware, no study has yet been conducted regarding the interrater agreement for the assessment of the leading edge of the bladder in PUS. Majida et al. [12], however, established the interrater agreement for the assessment of the bladder neck position. The resulting ICC, 0.61, indicated good agreement but is still inferior to the one calculated in the present study (ICC = 0.98). On the one hand, this is in accordance with the finding of our previous work that the bladder neck is sometimes difficult to assess during straining [10]. On the other hand, this might be due to the smaller sample of only 17 females. Alternatively, one should consider that Majida et al. used the reference line proposed by Dietz et al. [5], being sensitive to changes in the visional angle.

In comparison to the POP-Q system, our approach for PUS yielded better interrater agreement (*κ*-values) for the assessment of cystoceles. For POP-Q, Kobak et al. [13] found *κ* = 0.79, and we established *κ* = 0.98. One has to take into account, however, that the kappa found by Kobak et al. was calculated for the classification into the stages I to IV, whereas the kappa of the present study here was calculated for the classification “normal bladder and relevant cystocele” exclusively.

To our knowledge, for the interrater agreement of the assessment of cystoceles using MRI and DCP there are no studies available.

With regard to the clinical practice, we also determined the degree of agreement between POP-Q and PUS staging (normal bladder and relevant cystocele), which was good (*κ* = 0.65). This result is in accordance with former studies [5–7].

The surgical correction of cystoceles via an anterior colporrhaphy is mainly based on the restriction of the bladder mobility. As the three groups significantly differ in bladder mobility, different possible conclusions concerning the indication of surgical intervention might be drawn. For group I bladders, an intervention is most probably not helpful. For group III bladders, it will definitely reduce the cystocele. For group II bladders a surgical intervention might as well be indicated, but further clinical considerations should influence the decision.

The results of our study suggest that, due to its excellent interrater agreement and good correlation to POP-Q, PUS is a reliable tool for the clinical routine. The classification proposed here is furthermore able to give clinically relevant information and to help in the decision on the indication of a surgical intervention. Besides, PUS offers additional convenient features compared to POP-Q.

First of all, the imaging allows the doubtless identification of the nature of the prolapse. This is maybe the most important advantage PUS has to offer in contrast to POP-Q: hyperproliferative vaginal tissue or a prolapse of the vaginal vault can thus be distinguished from a true bladder prolapse, which is not possible using POP-Q.

Another very important characteristic of PUS is that its values are based on a fixed, bony structure, while POP-Q only uses soft tissue landmarks. If the initial position of the bladder should be regarded as physiologic or already pathologic is a question POP-Q does not have an answer to. Regarding women with loose pelvic floor structures, this is a doubtful basis for prolapse measurement.

Furthermore, PUS allows the dynamic evaluation of the pelvic floor during straining or coughing. Saving the volumes makes it possible to compare them to future scans after pelvic floor muscle training or after surgery in order to control the effectiveness of the chosen treatment.

Simultaneous evaluation of the urethra facilitates the diagnosis of comorbidities such as urethral kinking or funneling, obstruction, or paraurethral pathologies. The noninvasive nature of ultrasound is an advantage compared to the specula used in POP-Q assessment: they may prevent women from straining appropriately or cause a detorsion of the prolapse [14].

Our study is limited by the mean age of our study population, which is 58.2 years and thus not representative for females of all ages. As POP is not a pathology of young women, our values are a realistic reference for postmenopausal females. Furthermore, all of the patients originally are referred to the urogynecological department, mostly for symptoms of the lower urinary tract. The evaluation of younger women without any urogynecological symptoms might contribute to create basic reference values for the healthy female.

The presented classification needs a clinical evaluation including pre- and postoperative data in order to develop a predictive value needed for clinical routine.

In summary, the present study demonstrates that PUS and the introduced classification system are reliable tools for the evaluation of cystoceles. PUS and POP-Q show good correlation, as needed for the clinical routine. POP-Q remains the standard clinical diagnostic tool as it is helpful to identify the extent of prolapses in all compartments. According to the evaluation of the anterior prolapse, PUS seems to be a good additional tool for diagnostics as it shows the bladder position as the cause of the prolapsed and the complete urinary tract. Furthermore it is possible to compare dynamic images as needed to evaluate the success of treatment.

We recommend future studies to examine the clinical validity for the PUS in the diagnosis of cystoceles.

## Figures and Tables

**Table 1:** Means and standard deviations for bladder positions of groups I, II and III during rest and Valsalva and for the distance between rest and Valsalva position.

Means and standard deviations for groups I, II, and III					
	Group I	*p *(I versus II)	Group II	*p* (II versus III)	Group III
*P*_*R*_	3.08 ± 0.50	0.0012	2.60 ± 0.82	0.86	2.56 ± 0.56
*P*_*V*_	2.26 ± 0.55	<0.00001	0.61 ± 0.26	<0.00001	−0.90 ± 0.48
*D*_*RV*_	0.82 ± 0.49	<0.00001	2.00 ± 0.89	<0.00001	3.47 ± 0.70

Total	2.92 ± 0.61		1.51 ± 1.2		1.41 ± 1.14

*P*_*R*_: bladder position at rest; *P*_*V*_: bladder position at maximal Valsalva; *D*_*RV*_: distance from *P*_*R*_ to *P*_*V*_; *p* indicates the significance level of the established differences between groups I and II as well as between groups II and III.
